# Fluorescence Lectin Bar-Coding of Glycoconjugates in the Extracellular Matrix of Biofilm and Bioaggregate Forming Microorganisms

**DOI:** 10.3390/microorganisms5010005

**Published:** 2017-02-10

**Authors:** Thomas R. Neu, Ute Kuhlicke

**Affiliations:** Helmholtz Centre for Environmental Research – UFZ, 39114 Magdeburg, Germany; ute.kuhlicke@ufz.de

**Keywords:** biofilm, biofilm matrix, extracellular matrix (ECM), extracellular polymeric substances (EPS), lectin, fluorescence labelled lectin, glycoconjugate, laser microscopy, confocal microscopy, confocal laser scanning microscopy

## Abstract

Microbial biofilm systems are defined as interface-associated microorganisms embedded into a self-produced matrix. The extracellular matrix represents a continuous challenge in terms of characterization and analysis. The tools applied in more detailed studies comprise extraction/chemical analysis, molecular characterization, and visualisation using various techniques. Imaging by laser microscopy became a standard tool for biofilm analysis, and, in combination with fluorescently labelled lectins, the glycoconjugates of the matrix can be assessed. By employing this approach a wide range of pure culture biofilms from different habitats were examined using the commercially available lectins. From the results, a binary barcode pattern of lectin binding can be generated. Furthermore, the results can be fine-tuned and transferred into a heat map according to signal intensity. The lectin barcode approach is suggested as a useful tool for investigating the biofilm matrix characteristics and dynamics at various levels, e.g. bacterial cell surfaces, adhesive footprints, individual microcolonies, and the gross biofilm or bio-aggregate. Hence fluorescence lectin bar-coding (FLBC) serves as a basis for a subsequent tailor-made fluorescence lectin-binding analysis (FLBA) of a particular biofilm. So far, the lectin approach represents the only tool for in situ characterization of the glycoconjugate makeup in biofilm systems.  Furthermore, lectin staining lends itself to other fluorescence techniques in order to correlate it with cellular biofilm constituents in general and glycoconjugate producers in particular.

## 1. Introduction

The extracellular matrix of microbial biofilms constitutes an essential part of the biofilm structure. Nevertheless it represents a major issue in terms of characterization and dynamics in pure culture biofilms, in defined mixed culture biofilms, and especially in environmental biofilms. Consequently the matrix has been referred to as the dark matter of biofilms [1]. The techniques employed for biofilm matrix analyses are mainly classical extraction with subsequent chemical analysis (see table 2 in reference [2]), molecular characterization of cellular constituents versus extracellular space by genomic and proteomic approaches [3,4,5,6,7,8], and visualisation using various imaging techniques [9]. Due to its flexibility, laser scanning microscopy (confocal and two photon) became a standard technique for biofilm analyses (see table 2.1 in reference [1]). Furthermore multi-channel laser microscopy of hydrated biofilms has been used for imaging cellular and extracellular biofilm constituents with the option of recording additional signals such as reflection and autofluorescence [10]. Apart from proteins and extracellular DNA, the extracellular matrix of biofilms is mainly composed of polysaccharides [11,12]. Consequently, the application of probes specific for glycoconjugates, such as lectins, represents an obvious approach for in situ analyses of the hydrated biofilm matrix [13]. Screening of biofilm systems with the commercially available lectins by fluorescence lectin bar-coding (FLBC) has been used already in a number of studies [10,14,15,16,17,18,19,20] and served as the basis of subsequent fluorescence lectin-binding analysis (FLBA). Nevertheless, the actual screening data have not been published so far.

In this presentation, the detailed results of several lectin-screenings on a wide variety of samples are compiled and discussed. The image data sets recorded may be presented in the form of a binary bar-coding pattern, indicating no-binding and binding, as well as in the form of a heat map for the differentiation of the three levels of binding efficiencies. The lectin-binding data compiled in this report may serve as a guideline for selecting lectins in order to examine other microbial species.

## 2. Materials and Methods

### 2.1. Microbial Strains and Culture Conditions

The various species employed over the years, which were subjected to the lectin approach, are listed together with the growth medium or original citation (Table 1).

### 2.2. Lectin-Screening

Lectins were purchased from Sigma, EY Laboratories, Vector Laboratories, and Molecular Probes (see Supplementary Table S1). The lectins are usually available as fluorescein isothiocyanate (FITC), tetramethylrhodamine isothiocyanate (TRITC), Texas Red, or, more recently, as Alexa488 labelled conjugates. In case of unlabeled lectins the kit from Molecular Probes was used for labelling with Alexa488 by following the suppliers protocol. The lectins are usually supplied in solution at a 1 mg/mL buffer. From the stock solutions, aliquots were prepared for storage at −20 °C and were used at a dilution of 1:10 for staining. The details of biofilm lectin staining were reported previously [25,26].

Depending on the biofilm sample type, different staining and mounting techniques have to be employed. The various procedures of sample preparation for confocal laser scanning microscopy (CLSM) have been published in detail elsewhere [2]. Usually the hydrated (living) biofilm sample is covered with a few droplets of fluorescently labelled lectins and incubated for 20 min at room temperature in the dark. In case of paraformaldehyde (PFA) fixed samples, the PFA solution must be exchanged against a buffer. In a second step, the unbound lectins have to be removed by careful washing 3–4 times by using the appropriate liquid (filter sterilized river/tap water, buffer, or medium). The many options for washing different sample types were detailed in a protocol on biofilm matrix characterization [2]. For the screening, every lectin was applied as a single probe to an individual sample. Consequently the screening requires the identical number of samples matching the number of available lectins. A critical assessment of fluorescence lectin-binding analysis (FLBA) established the procedure, including optimal incubation time, lectin concentration, fluor conjugate, carbohydrate inhibition, order of addition, and lectin interaction [13].

For the examination, a wet mount was prepared by using one of; (1) a slide and coverslip, if needed with a spacer; or (2) a coverwell chamber with a defined spacer, both examined with a water immersion lens; or (3) mounting in a Petri dish and an examination with a water-immersible lens.

### 2.3. Epifluorescence and Laser Microscopy

The wet samples are first assessed by visual observation in the epifluorescence mode. Poor binding versus excellent binding can be easily distinguished by the appearance of the lectin signal. Dark (brownish) green means no binding, whereas bright green means good binding. If clear lectin-binding was observed visually, a sample data set in confocal mode was recorded. For this purpose the lookup table ‘glow-over-under’ (GOU) was used in order to optimize the signal-to-noise ratio. This routine assures very few saturated pixels and a background level close to zero. The details of CLSM instrument settings (major parameters) together with specific comments were compiled and saved in the form of a protocol in Excel (Microsoft).

For laser microscopy, a TCS SP1 and a TCS SP5X (Leica, Wetzlar, Germany) with an upright microscope were available. The SP1 system was equipped with traditional laser sources (Argon 488 nm, DPSS 561 nm, HeNe 633 nm) and controlled by the LCS software version 2.61. The SP5X system was equipped with a super continuum laser light source (470–670 nm) and controlled by the software LAS-AF ver. 2.4.1. Excitation of FITC and Alexa488 was done at 488 nm, and the emission signals were recorded from 500–550 nm.

### 2.4. Binary Bar-Coding and Heat-Mapping

For binary fluorescence lectin bar-coding (FLBC) the results were transferred into an excel sheet and color coded in black (binding) and white (no binding). For a more detailed presentation the various voltage settings of the photomultiplier (PMT) indicating sensitivity were translated into heat maps in order to differentiate three emission intensity ranges equal to lectin-binding efficiency. These were identical to the PMT voltage settings of 400–600 (strong signal), 600–800 (intermediate signal), and 800–1000 (weak signal).

### 2.5. Digital Image Analysis

Image data sets were projected employing the microscope software and Imaris ver. 8.3.1 (Bitplane). Images were printed from Photoshop CS6 (Adobe).

## 3. Results

### 3.1. Visual Examination

In order to apply lectins as a sensible probe for the detection of glycoconjugates in the biofilm matrix, a screening with all commercially available lectins should be performed. The procedure starts with a visual examination of the stained sample by epifluorescence microscopy. The judgement of lectin-binding can be easily done by the discrimination of faint brownish-greenish, spot-like green signal, bright green, and extended very bright green signals. Usually from the positive stains, a sample dataset is recorded at optimized imaging conditions using the lookup table glow-over-under (GOU). The signals should originate from or be associated with some kind of microbiological structure. Care must be taken in case of ‘artificial’, geometric, non-biological fluorescence, which could be autofluorescence of some origin, e.g., minerals or lectin binding to other particulate material.

### 3.2. Binary Bar-Coding

From the positive screening results (both bright green and very bright green), a simple binary bar-coding pattern can be generated, indicating a shortlist of lectins, which might be used for further analyses. This subsequent analysis, called fluorescence lectin-binding analysis (FLBA), can be employed for a more detailed investigation of different glycoconjugates within the extracellular matrix of biofilms. The lectins may be applied as single probes or in combination if conjugated with different fluorochromes. However, as lectins are usually glycoproteins, care must be taken to assure that the lectins do not bind to each other. This can be tested as a control by simply mixing the two lectins in solution on a slide and looking with epifluorescence microscopy for potential precipitates showing up in the intermediate fluor color. For example, two different lectins, e.g, with Alexa 488 (green emission) and Alexa 568 (orange/red emission), would result in a yellow precipitate.

### 3.3. Heat Map Bar-Coding

**Figure 1:**
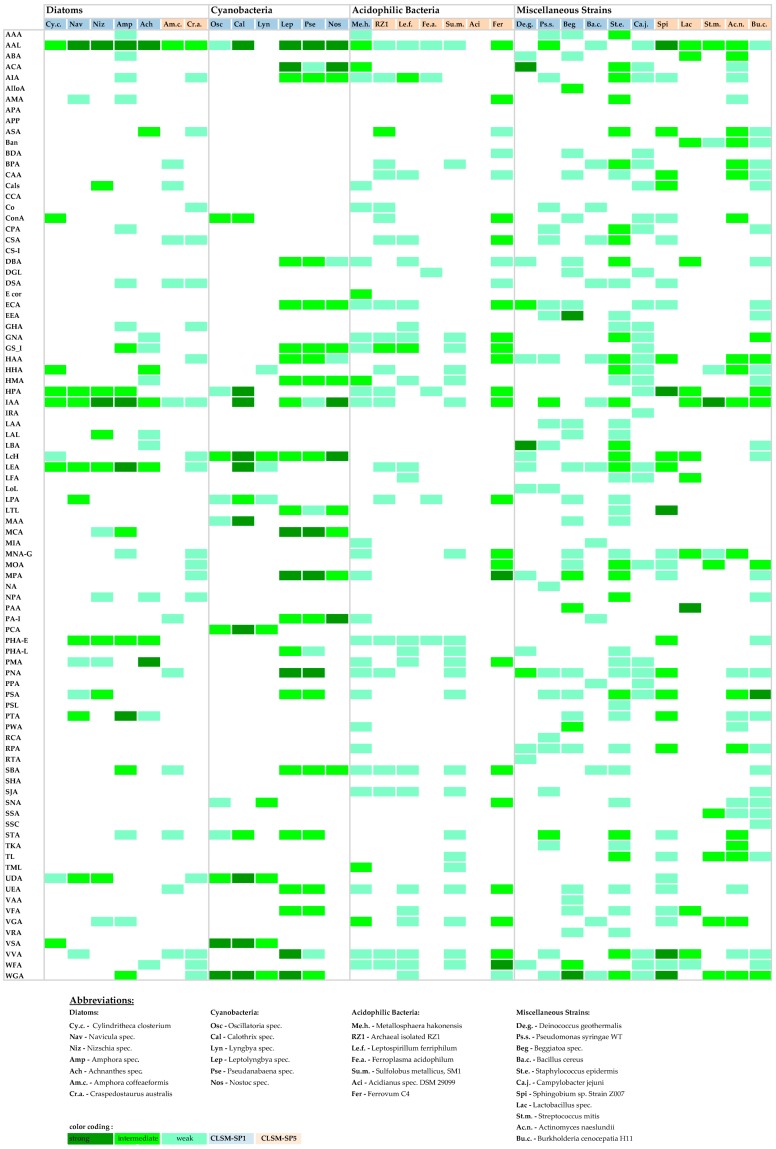
Fluorescence lectin bar-coding (FLBC) derived from confocal laser scanning microscopy and presented as heat map bar-coding pattern.

The positive binary bar-coding pattern can be further fine-tuned according to signal intensity. By simply using the sensitivity setting of the photomultiplier (PMT), the image data facilitates transfer into a heat map. The signal ranges were defined by the PMT settings at 400–600, 600–800, and 800–1000 voltage. Thus low sensitivity of the photomultiplier (voltage 400–600) means strong, intermediate sensitivity (voltage 600–800) means good, and high sensitivity (voltage 800–1000) means weak lectin binding. Thereby three different clusters of binding efficiency can be defined (Figure 1).

It has to be noted that, when using the lookup table glow-over-under (GOU) the results are optimal in terms of signal to noise ratio. However, as a consequence the images are similar in signal intensity although they are recorded at different photomultiplier sensitivities. Thus by careful examination, the image data sets of e.g., the weakly binding lectins show more noise due to the high PMT setting. In comparison, the efficiently binding lectins result in low noise images.

The heat map may be used similar to the binary bar-coding pattern described above in order to perform an even more detailed FLBA of a particular sample. Hence the heat map can be used to narrow down the shortlist of useful lectins. Furthermore it allows a reading across all lectins as well as across the various sample types. Thereby particular useful lectins may be identified which bind to the structural feature of interest.

### 3.4. Lectin-Binding Information

The lectin bar-coding patterns of the individual strains (Figure 1) are prone to counting. It allows the simple counting of positive signals, revealing a shortlist of favorite lectins in the order: (1) AAL, (2) IAA, (3) WGA, (4) VVA, (5) LEA, and (6) PNA. These lectins have specificities that seem to closely match the microbial glycoconjugates of the strains tested. The specificities in the same order are: (1) α-Fuc, (2) specificity not determined, (3) β-GlcNAc, (4) α-Man/α-GalNAc, (5) β-GlcNAc, and (6) β-Gal. More detailed results are compiled in Table 2, which shows 21 additional lectins with different binding efficiencies. Several of the lectins did not bind to any of the microbiological samples tested (APA, APP, CCA, CS-1, SHA).

The lectin pattern (Figure 1) may also be counted according to the number of lectins a particular strain has bound. For example, *Staphylococcus epidermis* bound overall 48 lectins, and *Burkholderia cenocepacia* bound 35 lectins. Other strains also showed a high binding efficiency, such as Sphingomonas and *Metallosphaera hakonensis*, both with 31 lectins bound, or Beggiatoa with 30 lectins bound. Figure 1 can be also be read with respect to the strains binding few lectins but with strong efficiency meaning high signal intensity. For example, Leptolyngbia showed 23 lectins with a strong signal, whereas Calothrix and Nostoc both had 13 lectins giving a strong signal. In contrast other strains showed reasonable lectin binding but overall only a low binding efficiency. Examples for this case are *Sulfolobus metallicus* and *Bacillus cereus*. Some strains tested did bind only very few lectins. Examples are *Ferroplasma acidophilum*, with only 6 lectins showing a signal.

**Figure 2:**
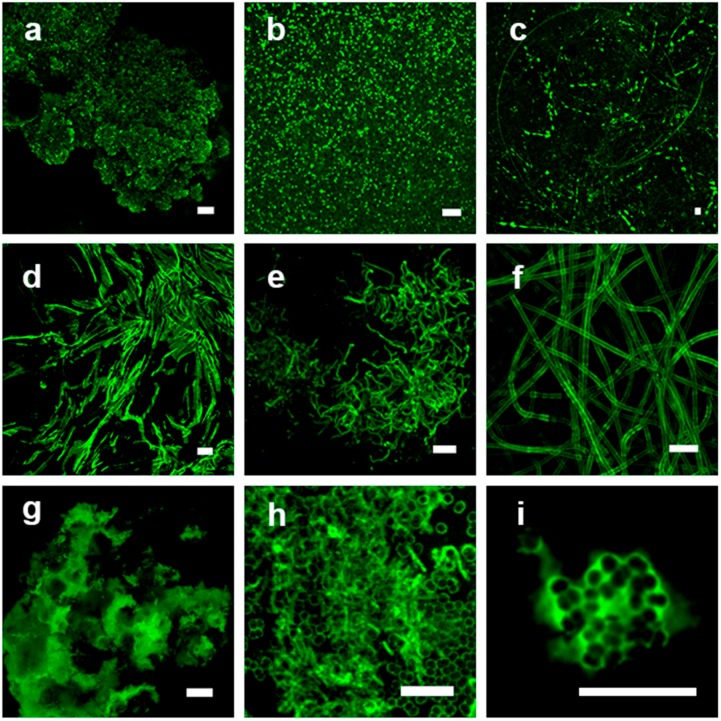
Confocal laser scanning microscopy of lectin stained microbiological samples representing a variety of binding patterns. The single-channel image data sets are shown as maximum intensity projection. (**a**) Cauliflower-like glycoconjugate distribution within a bio-aggregate, *Metallospaera hakonensis*; HMA-FITC; (**b**) cell surface signal from dense bacterial clusters, *Streptococcus mitis*; WGA-FITC; (**c**) glycoconjugate tracks of diatoms on a surface, *Craspedostaurus australis*; AAL-FITC; (**d**) slimy matrix in between filamentous microorganisms resulting in a negative staining, *Pseudanabaena* sp.; PHA-FITC; (**e**) cell surface of filamentous bacteria, *Sphingobium* sp.; WGA-FITC; (**f**) sheath of cyanobacteria filaments, *Leptolyngbia* sp.; LcH-FITC; (**g**) bio-aggregate with glycoconjugates showing partly a negative staining in non-binding regions, *Ferrovum* sp.; WFA-FITC; (**h**) capsule and rolling tracks of surface associated bacteria, *Deinococcus geothermalis*; PHA-FITC; (**i**) matrix signal of a microcolony, *Deinococcus geothermalis*; HAA-FITC. Scale bar = 10 µm.

The lectins are frequently applied in relation to a specific research question, meaning staining of a particular structural feature. For example, the lectins maybe employed for staining (1) microbial cell surfaces such as capsules or sheaths, (2) microbial footprints or holdfasts, (3) spatial or cloud-like structures of microcolonies, (4) microbe-microbe or microbe-eucaryote interactions, and (5) the overall matrix of biofilms or bio-aggregates. With this structural variety in mind, the results of binary and heat map bar-coding can be screened for particular lectin signals matching the target of interest. In Figure 2, examples for the different types of glycoconjugate appearances and structural patterns after lectin staining are presented.

## 4. Discussion

The fluorescence lectin bar-coding (FLBC) results compiled in this study may be presented as a binary bar-coding pattern (not shown) similar to a lectin microarray study using human cell lines [27]. The simple yes/no result in most cases is sufficient to select a panel of lectins for a more detailed analysis such as fluorescence lectin-binding analysis (FLBA). In addition, the results can also be depicted due to their fluorescence intensity. Thereby a bar-coding heat map can be generated which will allow a more detailed view of lectin-binding efficiency (Figure 1). Such a heat map was presented in a study on recombinant lectin microarrays for the analysis of human cells [28].

This preliminary summary of fluorescence lectin bar-coding (FLBC) results represents a basic set of glycoconjugate data for a particular range of pure culture samples. It may be used to narrow down the number of lectins applied to similar samples or as a guide for selecting lectins in different pure cultures. Nevertheless, in most cases, an individual lectin screening is necessary to assess the specific glycoconjugates produced in a particular sample under defined growth conditions. From lectin-barcoding combinations of lectins can also be selected for double or triple lectin staining if the appropriate controls are made. This will allow the identification of multiple glycoconjugate clusters in biofilms and bio-aggregate samples. In fact up to three different lectins carrying three different florescence labels have been combined for studying the glycoconjugate distribution in microcolonies of river biofilms [29,30].

After identifying a panel of binding lectins, these can be employed in various ways in order to examine the amount and quality of glycoconjugates produced. A nice example for applying a panel of lectins was reported for the multiple glycoconjugates produced by *Deinococcus geothermalis*. It could be shown that several of the glycoconjugates appear in different forms. They seem to function as a sticky adhesive to the substratum, some may have a role in surface movement, and others represent the connective component within the microcolonies produced [16]. Another example is bacterial cell surface glycoconjugates, with respect to mutants or closely related strains [15]. The approach was also employed for the assessment of microdomains in the polymer matrix of microcolonies. The triple lectin staining revealed three different matrix domains at the bacterial cell surface, in between the bacterial cells, and around the microcolonies [29,30]. In addition, the panel of selected lectins may be used for investigating the effect of growth conditions, nutrient composition and physicochemical factors on glycoconjugate production [31,32]. Lectins may be also employed for studying the effect of different chemicals (antibiotics, pharmaceutics, pesticides, etc.) on the glycoconjugate matrix [33,34]. In the latter two examples, the lectins allow characterization of the extracellular response against different environmental parameters. Thereby the lectin signal may serve as a parameter for characterizing the flux of matter and as a location for the sorption of organic and inorganic constituents.

Frequently the issue of controls for lectin binding, meaning lectin inhibition experiments, came up, which indeed represents a critical point [10]. Usually lectins have specificities for di- or oligosaccharides. However, for many lectins, the inhibiting sugar mentioned on the suppliers data sheet is a monosaccharide. Thus by using a monosaccharide for the inhibition of lectin-binding (having a di- or oligosaccharide specificity), a differential result may be obtained. The experience with several lectin controls showed (as expected) inhibition but also the enhancement of binding. This can be explained by the partial inhibition of the multiple lectin-binding sites, thereby creating a new specificity, which might result in a higher binding affinity. In any case, it should be clear that lectins are isolated from any kind of organism, usually plants, in which the lectin will find the perfectly matching biological binding site. In contrast, by applying lectins from another organism on a microbiological sample they are sort of ‘misused’ in a completely different biological system, in which the match for a specific binding site is unlikely or accidentally nearly fitting.

The question of how lectins bind to a particular structure has been raised in a detailed molecular study of lectin binding to structurally defined N-glycans [35]. By means of advanced NMR techniques, N-glycan/lectin interactions in solution were examined. It was found that epitope recognition depends on the structural context of both the carbohydrate and the lectin. In other words, the lectin may select distinct features of the target site in dependence on the presentation of the various carbohydrate residues and consequently cannot be assumed from solid-state interaction models. These findings are also crucial in our experiments, as the ‘misused’ lectins are frequently binding somehow to a non-target site.

For a critical assessment of the lectin approach, advantages and disadvantages have to be discussed. The main advantage is the commercial availability of lectins as probes having different specificities. Furthermore the lectins can be purchased with different fluorescence labels, or they may be custom labelled with an appropriate fluorochrome matching the sample properties. This will allow the application of different lectins in one sample as well as the combination of lectins with other fluorescent probes. By employing lectins, there is no need to produce antibodies against glycoconjugates, which in fact is rather difficult if compared to proteins. A disadvantage is the limited variation in specificity of the mainly plant-derived lectins. Another aspect lies in the ‘misuse’ of lectins in a microbiological system without a true biological binding site, which limits successful binding patterns. Despite these obstacles, control experiments are helpful for interpretation of the results [10]. Nevertheless fluorescence lectin bar-coding (FLBC) and fluorescence lectin-binding analysis (FLBA) will allow in most, but not all, cases in situ visualization and characterization of biofilm glycoconjugates.

Some of these obstacles were partly solved in other research areas by a new strategy. As already indicated, plant lectins suffer from availability, inconsistent activity, and the fact that they are often glycosylated, which hinders routine analysis of complex samples. This finally lead to the development of recombinant lectin microarrays [36]. By this approach, well-defined bacteria-derived lectins can be created with new specificities. This will dramatically enlarge the lectin library available and in the future ease the analysis of bacterial glycoconjugates.

At this point the application of lectin-microarrays has to be considered as well. The microarray technology was implemented in several variations using immobilized glycans or immobilized lectins, which were then tested against potential binding constituents, e.g., lectins or glycans [37,38,39,40]. Furthermore, lectin microarrays were also suggested for analysing the bacterial glycome [41,42]. The application of microarrays for studying the complex interactions of microorganisms with eukaryotic cells has been a compiled in two excellent reviews [43,44]. The high-throughput analysis allows examination of the bacterial glycosylation pattern and dynamic alteration of the carbohydrate coating. Thus, in contrast to the manual lectin approach with individual samples stained one by one with a particular lectin, the lectin microarray approach is undoubtedly faster. Nevertheless, both results might be rather similar. However, for positive results in the manual lectin approach, an image data set is usually recorded by laser microscopy. This represents an invaluable piece of information, which will facilitate structure-function studies. Consequently additional information is immediately available e.g., about the location and distribution of lectin binding at the bacterial cell surface, within a microcolony, across an aggregate, or throughout a biofilm. Another drawback of the microarray approach is its restriction to pure culture studies.

At this point the application of lectin-microarrays has to be considered as well. The microarray technology was implemented in several variations using immobilized glycans or immobilized lectins, which were then tested against potential binding constituents, e.g., lectins or glycans [37,38,39,40]. Furthermore, lectin microarrays were also suggested for analysing the bacterial glycome [41,42]. The application of microarrays for studying the complex interactions of microorganisms with eukaryotic cells has been a compiled in two excellent reviews [43,44]. The high-throughput analysis allows examination of the bacterial glycosylation pattern and dynamic alteration of the carbohydrate coating. Thus, in contrast to the manual lectin approach with individual samples stained one by one with a particular lectin, the lectin microarray approach is undoubtedly faster. Nevertheless, both results might be rather similar. However, for positive results in the manual lectin approach, an image data set is usually recorded by laser microscopy. This represents an invaluable piece of information, which will facilitate structure-function studies. Consequently additional information is immediately available e.g., about the location and distribution of lectin binding at the bacterial cell surface, within a microcolony, across an aggregate, or throughout a biofilm. Another drawback of the microarray approach is its restriction to pure culture studies.

Finally the application of other types of probes with specificities for glycoconjugates should be discussed. For example glycoconjugate-specific enzymes may represent a potential strategy for glycoconjugate detection. These enzymes are carrying a catalytic module as well as a carbohydrate binding module (CBM) [45,46]. Thus CBMs depict another class of probes for glycoconjugate characterization in hydrated biofilm systems. However, to the best of our knowledge, they have been used as a fluorescent probe for in situ glycoconjugate analysis by only one research group. The authors constructed a CBM-GFP fusion in order to examine the glycoconjugates in *E. coli* biofilms and bio-aggregates [47,48]. Interestingly CBMs were identified as part of extracellular microbial enzymes, where they facilitate binding to often un-accessible substrates [49,50]. Other potential probes are the many adhesins produced by microorganisms on their cell surface. These extensions of the cell surface in form of pili or fimbriae usually carry a lectin at their tip, which will facilitate highly specific binding to eucaryotic cell surfaces or to other microorganisms. Especially the latter ones involved in co-aggregation and co-adhesion [51,52,53], as well as those establishing host-microbe interactions (symbiotic, commensal or pathogenic) [54,55], are candidates for new lectins. These microbial cell surface lectins with their microbial-directed specificity would represent the ideal probe for microbiologically produced glycoconjugates in biofilm and bio-aggregates.

## 5. Conclusions and Perspective

1. Fluorescence lectin bar-coding (FLBC) represents a powerful approach for selecting a panel of lectins useful for subsequent fluorescence lectin-binding analysis (FLBA).
2. The time needed for lectin bar-coding is dependent on the difficulty in mounting the samples. It will also depend on the number of lectins bound, which is equal to the number of datasets to be recorded.
3. Usually lectin bar-coding on one sample type with the current range of commercially available lectins (≈70) can be easily performed within two days.
4. Subsequent fluorescence lectin-binding analysis (FLBA) with a selected panel of positive lectins is often combined with nucleic acid staining. This allows visualization of bacterial cell and glycoconjugate distribution as well as lectin-specific glycoconjugate typing see [16].
5. FLBA may be combined with fluorescence in situ hybridisation (FISH) [56] or catalized reporter deposition fluorescence in situ hybridisation (CARD-FISH) [18]. Thereby the glycoconjugate signal can be related to a defined bacterial species or to phylogenetic groups of bacteria.
6. The advantage of lectin bar-coding as compared to microarray techniques lies in the immediate visual information and identification of the lectin binding pattern.

## Figures and Tables

**Table 1:** Strains examined by fluorescence lectin bar-coding (FLBC).

Strain	Medium/Growth Conditions/Reference
Various diatoms	ASW plus inorganic nutrients, liquid culture
*Amphora caffeaeformis*	[21]
*Craspedostauros australis*	[21]
Various cyanobacteria	BG 11, liquid culture
*Metallosphaera hakonensis*	DSMZ 88 medium, 65 °C, pH 2.5, grown on 10 µm chalcopyrite grains in Erlenmeyer flasks
*Leptospirillum ferriphilum*	MAC, 0.02 % yeast extract, pH 1.8, pyrite grains in Erlenmeyer flasks
*Acidianus* sp. DSM 29099	[20]
*Ferroplasma acidiphilum* DSM 29986	[20]
*Sulfolobus metallicus* DSM 6482	[20]
Ferrovum C4	iFeo medium, pH 2.5, liquid culture
*Deinococcus geothermalis*	[16]
*Pseudomonas syringae*	[15]
Beggiatoa	[22]
*Bacillus cereus* 10987	TSB + 0.5% yeast extract, IBIDI 96 well microplates
*Streptococcus epidermidis* 1457	TSB, IBIDI 96 well microplates
*Campylobacter jejuni*	[23]
*Sphingobium* sp. Z007	[24]
*Lactobacillus* sp.	THB, liquid culture, 35 °C
*Streptococcus mitis*	THB, liquid culture, 35 °C
*Acinetobacter naesludii*	THB, liquid culture, 35 °C
*Burkholderia cenocepacia* H111	NYG, YEB, membrane filters on agar plates

**Table 2:** Shortlist of lectins that bound most often to the species used in this study. The lectins are listed in the order of binding frequency.

Lectin	Specificity
AAL	α-Fuc
IAA	n. d.
WGA	β-GlcNAc
VVA	α-Man, α-GalNAc
LEA	β-GlcNAc
PNA	β-Gal
AIA	n.d.
ECA	α-Gal, β-Gal, α-GalNAc, β-GalNAc
HAA	α-GlcNAc, α-GalNAc
HPA	α-GalNAc
PSA	α-Man, α-Glc, α-GlcNAc
SBA	α-GalNAc, β-GalNAc
WFA	α-GalNAc, β-GalNAc
DBA	α-GalNAc
LcH	α-Man, α-Glc, α-GlcNAc
MNA	n.d.
PHA-E	n.d.
STA	β-GlcNAc
UEA	β-GlcNAc, α-Fuc
GS-I	α-Gal, α-GalNAc
HHA	α-Man
MPA	n.d.
PMA	n.d.
VGA	n.d.
ConA	α-Man, α-Glc, α-GlcNAc
GNA	α-Man
HMA	α-Fuc, α-GalNAc

n.d.—lectin specificity is not yet determined.

## Supplementary Materials

The following are available online at www.mdpi.com/2076-2607/5/1/5/s1, Table S1: Overview of lectins available, sorted according to their three-letter code. (Table with the lectins used in this study that are commercially available. The table shows the Latin and common name of the organism from which the lectin was isolated, as well as the three letter abbreviation of the lectin isolate. In addition, the fluorochrome labels and lectin specificities are listed.)

## Abbreviations

The following abbreviations are used in this manuscript:

DSM: Deutsche Sammlung für Mikroorganismen
DSMZ: Deutsche Sammlung für Mikroorganismen und Zellkulturen
ASW: artificial seawater
TSB: tryptic soy broth
THB: Todd Hewitt broth
MAC: Mackintosh basal salt solution
NYG: nutrient broth supplemented with yeast extract and glucose
YEB: complex medium with yeast extract, beef extract, peptone and sucrose
Fuc: Fucose
Gal: Galactose
GalNAc: N-Acetyl galactosamine
Glc: Glucose
GlcNAc: N-Acetyl glucosamine
Man: Manose

Abbreviations for microorganisms are listed in Figure 1.

The three letter code for lectins is explained in Supplementary Table S1.
