# Supplementary material for: Fluorescence Lectin Bar-Coding of Glycoconjugates in the Extracellular Matrix of Biofilm and Bioaggregate Forming Microorganisms
Source: Microorganisms. 2017 Feb 10;5(1):5. doi: 10.3390/microorganisms5010005 (PMC5374382; doi:10.3390/microorganisms5010005)
Supplement: Supplementary file 1 [file microorganisms-05-00005-s001.pdf]

Supplementary Table 1: Overview of lectins available, sorted according to their three-letter code.

Nr.	Lectin			Fluo label	Inhibiting carbohydrate	Linkage type	Supplier[1)]
	Latin name	Common name	3-letter code				
1	*Anguilla anguilla*	fresh water eel	AAA	FITC	α-Fuc		EY Labs
2	*Aleuria aurantia*	mushroom	AAL	Alexa488, Fluores.	α-Fuc	Fuc(α1,6)GlcNAc, Fuc(α1,3)GalNAc, Fuc(α1,2)Gal(β1,4)[Fuc(α1,3/4)]Gal(β1,4)GlcNAc	**Vector Labs**
3	*Agaricus bisporus*	mushroom	ABA	FITC	α-Gal	Gal(β1,3)GalNAc	EY Labs
4	*Amaranthus caudatus*	Inka wheat	ACA	FITC		Gal(β1,3)GalNAc	EY Labs
5	*Artocarpus integrifolia*	jackfruit	AIA	FITC	β-Gal	Gal(β1,3) GalNAc	EY Labs
6	*Allomyrina dichotoma*	Japanese beetle	AlloA	FITC	β-Gal		
7	*Arum maculatum*	lords and ladies tubers	AMA	FITC		Man	EY Labs
8	*Aegopodium podagraria*	ground elder	APP	FITC	α-GalNAc, β-GalNAc	not determined	EY Labs
9	*Allium sativum*	garlic	ASA	FITC	α-Man	D-Man(α1,3)	EY Labs
10	*Musa paradisiaca*	banana	Ban	Fluores.	Glc, Man	(α 1,3) glucosyl- and mannosyl- residues	Vector Labs
11	*Bryonia dioica*	white bryony	BDA	FITC	α-GalNAc, β-GalNAc	not determined	EY Labs
12	*Bauhinia purpurea*	camel's foot tree	BPA	FITC	α-GalNAc, β-GalNAc		EY Labs
13	*Colchicum autumnale*	autumn crocus	CA	FITC		not determined	EY Labs

14	*Caragana aborescens*	pea tree	CAA	FITC	α-Gal, β-Gal, α-GalNAc, β-GalNAc		EY Labs
15	*Calystega sepiem*	hedge bindweed	Calsepa	FITC		Man>Glc>Fuc>GlcNAc	EY Labs
16	*Cancer antennarius*	California crab	CCA	Alexa488	sialic acid	9-O-Ac-NeuAc>4-O-Ac-NeuAc	**EY Labs**
17	*Codium fragile*	green sea fingers	Co	Alexa488	GalNac		**Sigma**
18	*Canavalia ensiformis*	jack bean	ConA	FITC	α-Man, α-Glc, α-GlcNAc	branched Man	Sigma
19	*Cicer arietinum*	chick pea	CPA	FITC		Not determined	EY Labs
20	*Cytisus scoparius*	Scotch broom	CSA	FITC	β-Gal	lactose	EY Labs
21	*Dolichos biflorus*	horse gram	DBA	FITC	α-GalNAc	GalNAc(α1,3)GalNAc	Sigma
22	*Dioclea grandiflora*		DGL	FITC	Man, Glc		EY Labs
23	*Datura stramonium*	jimson weed	DSA	FITC	β-GlcNAc	GlcNAc(β1,4)GlcNAc oligomers, Gal(β1,4)GlcNAc	EY Labs
24	*Erythrina cristagalli*	cockspur coral tree	ECA	FITC	α-Gal, β-Gal, α-GalNAc, β-GalNAc	Gal(β1,4)GlcNAc	EY Labs
25	*Erythrina corallodendron*	coral tree	Ecor	Alexa488	Gal, GalNAc		**Sigma**
26	*Euonymus europaeus*	spindle tree	EEA	FITC		Gal(α1,3)[Fuc(α1,2)] Gal	EY Labs
27	*Glechoma hederacea*	ground ivy	GHA	FITC	GalNAc		EY Labs
28	*Galanthus nivalis*	snowdrop	GNA	FITC	α-Man	Man(α1,3)Man	EY Labs
29	*Griffonia simplicifolia*		GS-I	FITC	α-GalcNAc, α-Gal		Sigma
30	*Helix aspersa*	garden snail	HAA	FITC	α-GlcNAc, α-GalNAc	not determined	EY Labs

31	*Hippeastrum hybrid*	amaryllis	HHA	FITC	α-Man	Man(α1,3) Man(α1,3) Man(α1,3) Man(α1,2)Man	EY Labs
32	*Homarus americanus*	California lobster	HMA	Alexa488, FITC	Sialic Acid, α-Fuc, α-GalNAc		EY Labs
33	*Helix pomatia*	edible snail	HPA	FITC	α-GalNAc	not determined	Sigma
34	*Iberis amara*	candy tuft	IAA	Alexa488		not determined	**EY Labs**
35	*Iris hybrid*	dutch Iris	IRA	FITC			EY Labs
36	*Laburnum alpinum*	Scotch alburnum	LAA	FITC	β-GlcNAc	GlcNAc(β1,4)GlcNAc	EY Labs
37	*Laburnum anagyroides*	golden chain/rain	LAL	FITC	α-Me-L-Fuc	Fuc(α1,2)Gal(β1,4)Glu and Fuc(α1,2)Ga(β1,4)Glc(β1,6)GalNAc[(β1,3) Gal]	EY Labs
38	*Phaseolus lunatus*	lima bean	LBA	FITC	α-GalNAc	GalNAc(α1,3)[Fuc-(α1,2)]Gal	EY Labs
39	*Lens culinaris*	lentil	LcH	FITC	α-Man, α-Glc, α-GlcNAc	branched Man with α-Fuc as determinant	EY Labs
40	*Lycopersicon esculentum*	tomato	LEA	FITC	β-GlcNAc	GlcNAc(β1,4)GlcNAc oligomers	Sigma
41	*Limax flavus*	garden slug	LFA	FITC	sialic acid		EY Labs
42	*Tetragonolobus purpurea*	asparagus pea	Lotus	FITC	α-Fuc	Fuc(α1,2)Gal(β1,4)[Fuc (α1)]GlcNAc	Sigma
43	*Limulus polyphemus*	horseshoe crab	LPA	FITC	sialic acid		EY Labs
44	*Maackia amurensis*	maackia	MAA	FITC	sialic acid, Gal		EY Labs
45	*Mangifera indica*	mango	MIA	Alexa488			**EY Labs**
46	*Morniga G*	black mulberry	MNA-G	FITC		Gal	EY Labs

47	*Marasmius oreades*	mushroom	MOA	FITC		Gal(α1,3)Gal, Gal(α1,3)Gal(β1,4)GlcNAc/Glc	EY Labs
48	*Maclura pomifera*	osage orange	MPA	FITC	α-Gal, α-GalNAc	Gal(β1,3)GalNAc	EY Labs
49	*Narcissus pseudonarcissus*	daffodil	NPA	FITC		Not determined	EY Labs
50	*Perseau americana*	avocado	PAA	Alexa488			**EY Labs**
51	*Pseudomonas aeruginosa*		PA-I	Alexa488	Gal		**Sigma**
52	*Phaseolus vulgaris*	red kidney bean	PHA-E	FITC		Gal	EY Labs
53	*Phaseolus vulgaris*	red kidney bean	PHA-L	FITC		Gal, GlcNAc, Man	EY Labs
54	*Polygonatum multiflorum*	solomon`s seal	PMA	FITC		Man	EY Labs
55	*Arachis hypogaea*	peanut	PNA	FITC	β-Gal	Gal(β1,3)GalNAc	EY Labs
56	*Ptilota plumosa*	red marine algae	PPA	Alexa488			**Sigma**
57	*Pisum sativum*	garden pea	PSA	FITC	α-Man, α -Glc, α-GlcNAc	branched Man with α-Fuc as determinant	Sigma
58	*Polyporus squamosus*	mushroom	PSL	FITC		Neu5Ac(α2,6)Gal(β1,4)GlcNAc	EY Labs
59	*Psophocarpus tetragonolobus*	winged bean	PTA	FITC	β-Gal, α-GalNAc, β-GalNAc	Gal	EY Labs
60	*Phytolacca americana*	pokeweed	PWA	FITC	β-GalNAc	GlcNAc(β1,4)GlcNAc oligomers and [Gal-(β1,4)lcNAc]$_2$	EY Labs
61	*Robinia pseudoaccacia*	black locust	RPA	FITC		not determined	EY Labs
62	*Ricinus communis*		RCA	Fluo	Gal, GalNAc		Vector Labs
63	*Trifolium repens*	white clover	RTA	Alexa488			**EY Labs**
64	*Glycine max*	soybean	SBA	FITC	α-GalNAc, β-GalNAc	GalNAc(α1,3)Gal	EY Labs
65	*Sophora japonica*	Japanese pagoda tree	SJA	FITC	β-GalNAc	GalNAc(β1,6)Gal	EY Labs

66	*Salvia horminum*		SHA	Alexa488	GalNAc		EY Labs
67	*Sambucus nigra*	elderberry	SNA	Fluo	β-Gal, sialic acid	(α2,6)Gal, GalNAc	EY Labs
68	*Salvia sclarea*		SSA	FITC	GalNAc		EY Labs
69	*Sarothamnus scoparius*		SSC	Alexa488			EY Labs
70	*Solanum tuberosum*	potato	STA	FITC	β-GlcNAc	GlcNAc(β1,4)GlcNAc oligomers	EY Labs
71	*Trichosanthes kirilowii*	Chinese cucumber	TKA	FITC	β-Gal	lactose	EY Labs
72	*Tulipa sp*	tulip	TL	FITC	α-GalNAc, β-GalNAc	GalNAc, Gal, Fuc	EY Labs
73	*Tritrichomonas mobilensis*		TML	Alexa488			not available anymore
74	*Urtica dioica*	stinging nettle	UDA	FITC	β-GlcNAc	not determined	EY Labs
75	*Ulex europaeus*	gorse or furze	UEA	FITC	β-GlcNAc, α-Fuc	L-Fuc(α-1,2)Gal(β1,4)-GlcNAc	Sigma
76	*Vicia faba*	fava bean, broad bean	VFA	FITC	α-Man, α -Glc, α-GlcNAc	branched Man with α-Fuc as determinant	EY Labs
77	*Vicia graminea*		VGA	Alexa488, FITC		O-linked Gal(β1,3)GalNAc	EY Labs
78	*Vigna radiata*	mung bean	VRA	FITC	α-Gal		EY Labs
79	*Vicia villosa*	hairy vetch	VVA	FITC	α-Man; α-GalNAc	Man, GalNAc(α1,3)Gal	EY Labs
80	*Wisteria floribunda*	japanese wisteria	WFA	FITC	α-GalNAc, β-GalNAc	GalNAc(α1,6)Gal	EY Labs
81	*Triticum vulgaris*	wheat	WGA	FITC	β-GlcNAc; Sialic acid	GlcNAc(β1,4)GlcNAc	Sigma

[1)] self-labelled Alexa488 lectins printed in bold
